# An evaluation of DistillerSR’s machine learning-based prioritization tool for title/abstract screening – impact on reviewer-relevant outcomes

**DOI:** 10.1186/s12874-020-01129-1

**Published:** 2020-10-15

**Authors:** C. Hamel, S. E. Kelly, K. Thavorn, D. B. Rice, G. A. Wells, B. Hutton

**Affiliations:** 1 Clinical Epidemiology Program, Ottawa Hospital Research Institute, 501 Smyth Road, Box 201b, Ottawa, Ontario K1H 8L6 Canada; 2 Department of Medicine, University of Split, Split, Croatia; 3 Cardiovascular Research Methods Centre, University of Ottawa Heart Institute, Ottawa, Ontario Canada; 4 School of Epidemiology and Public Health, University of Ottawa, Ottawa, Ontario Canada; 5 Department of Psychology, McGill University, Montreal, Quebec Canada

**Keywords:** Artificial intelligence, Systematic reviews, Rapid reviews, Prioritization, Automation, Natural language processing, Machine learning, Time savings, Efficiency, True recall

## Abstract

**Background:**

Systematic reviews often require substantial resources, partially due to the large number of records identified during searching. Although artificial intelligence may not be ready to fully replace human reviewers, it may accelerate and reduce the screening burden. Using DistillerSR (May 2020 release), we evaluated the performance of the prioritization simulation tool to determine the reduction in screening burden and time savings.

**Methods:**

Using a true recall @ 95%, response sets from 10 completed systematic reviews were used to evaluate: (i) the reduction of screening burden; (ii) the accuracy of the prioritization algorithm; and (iii) the hours saved when a modified screening approach was implemented. To account for variation in the simulations, and to introduce randomness (through shuffling the references), 10 simulations were run for each review. Means, standard deviations, medians and interquartile ranges (IQR) are presented.

**Results:**

Among the 10 systematic reviews, using true recall @ 95% there was a median reduction in screening burden of 47.1% (IQR: 37.5 to 58.0%). A median of 41.2% (IQR: 33.4 to 46.9%) of the excluded records needed to be screened to achieve true recall @ 95%. The median title/abstract screening hours saved using a modified screening approach at a true recall @ 95% was 29.8 h (IQR: 28.1 to 74.7 h). This was increased to a median of 36 h (IQR: 32.2 to 79.7 h) when considering the time saved not retrieving and screening full texts of the remaining 5% of records not yet identified as included at title/abstract. Among the 100 simulations (10 simulations per review), none of these 5% of records were a final included study in the systematic review. The reduction in screening burden to achieve true recall @ 95% compared to @ 100% resulted in a reduced screening burden median of 40.6% (IQR: 38.3 to 54.2%).

**Conclusions:**

The prioritization tool in DistillerSR can reduce screening burden. A modified or stop screening approach once a true recall @ 95% is achieved appears to be a valid method for rapid reviews, and perhaps systematic reviews. This needs to be further evaluated in prospective reviews using the estimated recall.

## Background

Systematic reviews (SRs) aim to minimize bias by using systematic and rigorous methods [1]. This process, however, can require substantial resources (e.g., cost and humans), and in some cases can require more than 12 months to complete. An analysis of 195 reviews registered in PROSPERO reported a mean time (from registration to publication) of 67.3 weeks (standard deviation 31 weeks, range 6 to 186 weeks) and a mean author team of 5 people [standard deviation (SD): 3, range 1 to 27 people] [2].

It is not uncommon for a systematic search to yield a large number of records, many of which are irrelevant (i.e., low precision) [2, 3]. In a recent study, of 139,467 citations among 25 reviews, 5.48% (95% confidence interval (CI) 2.38 to 8.58%) of the citations were included in the final reviews [3]. Such volume introduces opportunity for human error in the screening process [3–5]. While screening of titles and abstracts represents only one step in the series of tasks involved in the conduct of SRs, due to the high screening burden, the resources for this step can be a large proportion of the total human resource time spent on the review [6]. Several strategies have been evaluated to decrease time spent screening titles and abstracts, including the use of dual monitors for screening [7], title only screening [8], a staged title only followed by abstract screening [6], screening by one reviewer [5, 9–12], and using artificial intelligence (AI) tools (e.g., text mining, prioritization) [11, 13–17].

Several software tools exist that support title and abstract screening in SRs [18], however not all packages currently include the capacity to implement machine learning techniques for citation screening [19]. Among those that do, there is variation in the level of sophistication of the machine learning tool, the algorithms used, the cost of the software package, and if and how often it is updated and supported. The most commonly evaluated software are Abstrackr, DistillerSR, EPPI-Reviewer, RobotAnalyst, SWIFT-Active Screener, and SWIFT-Review [13–16, 20–24], with varying success depending on the size of the datasets, the machine learning algorithm, and the level of replacement of humans with AI [25]. While AI may not be ready to fully replace human screeners in the task of study selection, studies suggest that optimizing, accelerating, and reducing screening burden through the use of AI-informed screening methods represents a viable option. This includes *prioritized screening*, where the presentation of titles and abstracts to reviewers is continually adjusted, through active machine learning, based on the AI’s estimated likelihood of relevance [17]. In circumstances of present day where the requestors (end users) of a particular knowledge synthesis frequently are in search of a rapidly generated synthesis of the available evidence for a research question of interest, such tools may offer attractive gains to research teams if safely implemented to minimize the risk of falsely excluding relevant evidence.

A 2015 systematic review concluded that there is almost no replication between studies or collaboration between research teams evaluating text mining methods, which makes it difficult to establish overall conclusions about best approaches [17]; this represents an especially troublesome barrier toward wider adoption of the use of such methods globally in knowledge syntheses. Another important barrier to uptake for many research teams is uncertainty as to the proper set-up and implementation, both in terms of settings within the software as well as incorporation into the well-established SR process.

### Objectives

Using the AI simulation tool (which uses the prioritization algorithm) in DistillerSR, the primary objectives of this study were to:

1. Empirically evaluate the reduction in *screening burden* (the number of records not required to be screened) once a *true recall @ 95%* was achieved (i.e., once 95% of the studies included based on the title/abstract to be further evaluated based on the full-text were identified).
2. Evaluate the performance using a true recall @ 95%. Specifically, to identify if any of the studies that were included in the systematic review were among the 5% of *records that were not yet identified* as included based on the title/abstract [i.e., *title/abstract false negatives (FN)*].

We chose DistillerSR software (Evidence Partners Incorporated; Ottawa, Canada), as it is amongst the most widely used systematic review management software programs worldwide, and because our research teams are long-time users of this software. A list of terminology (italicized terms) used in the manuscript with descriptions are provided in Table 1.

**Table 1:** Terminology and descriptions

Terminology	Description
Estimated recall	The estimated percent of how many studies at title/abstract level have been identified among those that will be passed through to full-text screening. As this is calculated based on a set of records that have not been completely screened, the estimated recall may differ from the true recall.
Final include	A primary study included in the completed systematic review.
Iteration	A set of records that is used to assign a score around the likeliness of inclusion and prioritize the remaining unscreened records in order from highest relevance to lowest relevance.
Modified screening approach	An approach to modify how screening is being performed. For example, changing from: (i) dual-independent screening to liberal accelerated screening; (ii) dual-independent screening to single-reviewer screening; or (iii) assigning the remaining records to the AI reviewer to exclude, with a human reviewer(s) also screening these records as a second reviewer.
Prioritized screening	Through active machine learning, the presentation of records to reviewers is continually adjusted based on the AI’s estimated likelihood of relevance. The frequency of adjustment may differ by software application.
Screening burden	The total number of records at title/abstract to be screened.
Stop screening approach	An approach to screening whereby the remaining records are not screened once a certain threshold has been achieved (e.g., estimated recall @ 95%). These records are assumed to be excluded.
Record not yet identified [i.e., title/abstract false negative (FN)]	When an estimated recall (at any %) or true recall of less than 100% is used, these are the records that would have been included based on the title/abstract to be further reviewed at full-text screening, but were not yet identified. Had these records been screened at title/abstract and further screened based on the full text, they may have been excluded or included in the final review (i.e., a final include).
Title/abstract include [i.e., title/abstract true positive (TP)]	Records included based on the title/abstract to be further reviewed based on the full text. These records may then be excluded at full-text review or included in the final review.
Training set	One or more iterations which inform the machine learning to score and prioritize the remaining unscreened records.
Title/abstract exclude [i.e., true negative (TN)]	Records considered excluded based on title/abstract screening.
True recall	This is only known once all references have been screened and includes the percentage of the actual number of records that were title/abstract includes. True recall % calculated as: [title/abstract TP / (title/abstract TP + title/abstract FN)]

There is currently no agreed upon *modified screening* or *stop screening* approach where a review team may decide to modify how records are being screened (e.g., changing from dual-independent screening to single-reviewer screening) or stop screening the remaining records. For the current study, we are evaluating a true recall @ 95%. In other words, once the AI simulation tool has identified 95% of the studies that were included based on the title/abstract to be further reviewed based on the full text [i.e., *title/abstract true positives (TP*)], we would assign the AI reviewer to exclude the remaining studies which would include approximately 5% of the title/abstract records that were included but not yet identified (i.e., title/abstract FP) and the title/abstract excludes [i.e., *true negatives (TN*)]. This number (95%) was selected as it is a common recall number used when measuring the reduction in workload; it also approximates the level of human error in screening [3, 16, 26]. Therefore, true recall @ 95% is calculated as [title/abstract TP / (title/abstract TP + title/abstract FN)]. The distinction between true recall and *estimated recall* (as would be calculated in a prospective review) is that, as we used completed reviews, we know the actual number of studies that were included based on the title/abstract screening to be further evaluated based on the full text [23]. The findings from this study will help toward establishing the validity of this approach to citation screening as a potential additional source of time savings in the context of conducting systematic reviews and other knowledge synthesis products, including rapid reviews [27–30] and living systematic reviews [31, 32]. Furthermore, given that challenges in set-up are a known barrier amongst knowledge synthesis teams toward the decision to implement machine learning methods for their research [25], a secondary objective of the study was to provide transparent, repeatable methods for other review teams to replicate in their own research. This will allow for further testing of this process, thereby increasing the sample size and external validity of the results presented in this study.

### Study methods

The protocol for this study was registered on the Open Science Framework (OSF: https://osf.io/2fgz7/) and was conducted using the AI simulation module within DistillerSR Software (May 2020 release). This version (2.31.0) of DistillerSR has fully replaced all existing AI functionality from earlier versions and includes prioritized reference screening (i.e., re-sorting records at regular screening intervals based on the AI tool’s estimated probability of relevance for each remaining record) and the development of a system in which to create custom classifiers [e.g., automatically labeling randomized controlled trials (RCTs)].

This study used information from 10 previously completed SRs (i.e., responses to screening at title/abstract and the final list of included studies) that were undertaken by research teams that perform a high volume of knowledge synthesis reviews, led by our co-authors, located at the Ottawa Hospital Research Institute and the University of Ottawa Heart Institute in Ottawa, Canada. We selected 10 reviews in this pilot experiment to capture a variety of topic areas, review sizes, and inclusion rates. An overview of the characteristics of these reviews, with brief descriptions of the objectives and PICO elements (participants, interventions, comparators, outcomes) is provided in Additional file 1.

Methods on how we implemented DistillerSR’s AI simulation tool for citation screening have been described in detail in Additional file 2 for researchers who are interested in running simulations using their own review projects. In the context of the current study, DistillerSR’s AI simulation tool selects a random set of records which contains 2% of the dataset (with a minimum of 25 records and a maximum of 200 records). Each set of these records is called an *iteration*. and the simulation tool uses the responses already provided (title/abstract included and excluded responses, based on our previous SRs) to build the first iteration (i.e., the initial *training set*). Subsequently, the remaining unscreened records are assigned a score (by the software) relating to the likelihood of inclusion, and references are re-ranked (i.e., prioritized) in order of this score (from most to least likely to be relevant). The next iteration (i.e., the next 2% of the records) is then run, and all remaining records are assigned an updated score based on the likelihood of inclusion estimated using the information gathered from all iterations, which creates the newest training set. This process continues until all records are screened. The AI simulation tool mimics the process of human screening. In a prospective review, responses from the reviewers would be used to build the iterations (e.g., using single reviewer, dual independent review with conflicts resolved), but would otherwise function in the same manner. Once prioritization is set up (i.e., one click when managing levels), the process of prioritization occurs automatically in the background without intervention from the reviewers, making it easy to use, and thereby providing the potential to identify relevant literature more efficiently.

**Fig. 1:**
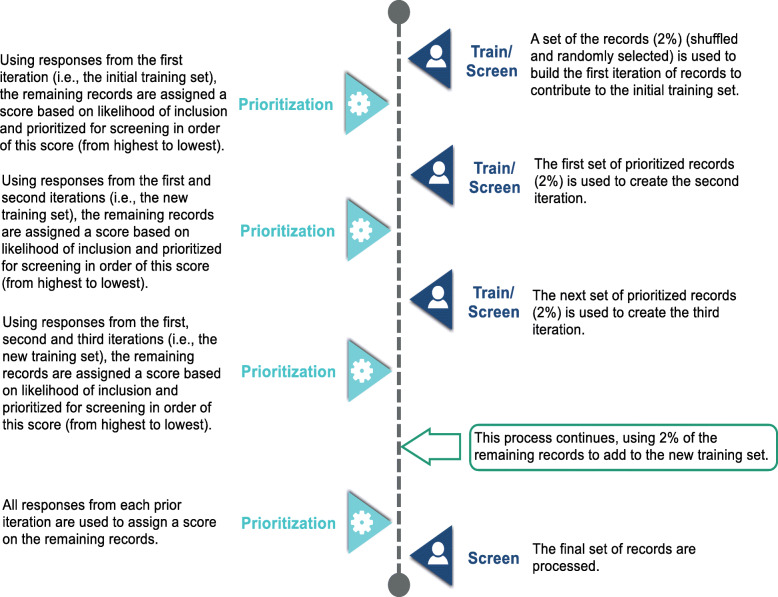
AI simulation flow

Figure 1 represents how the simulation tool uses the existing information (i.e., responses) to simulate the performance of the prioritization tool.

### Data collection

For each of the 10 SRs that served as experimental units for this work, we ran the AI simulation 10 times to account for any variation in the simulations, and to introduce randomness (through shuffling the references, which is automatically performed by the software) into the initial training sets. After each simulation was run, the following information was recorded at the first iteration that identified 95% of the studies included from title/abstract to be further evaluated at full text (i.e., true recall @ 95%):

- *The number of records per iteration and the number of iterations*. An iteration contains 2% of the total number of records, with a minimum of 25 and a maximum of 200 records per iteration. This allowed for measuring the variation within a review around the number of records at title/abstract not yet identified (i.e., title/abstract FN).
- *The total number of records screened (*i.e.*, screening burden).* This is composed of 95% of the title/abstract included studies and a portion of the title/abstract excluded studies.
  Calculation: (title/abstract TP + title/abstract TN).
- *The number of records included at title/abstract to be further reviewed at full-text screening once a true recall @ 95% was achieved* (title/abstract TP). This could account for slightly more than 95% of the studies, depending on the how many of these studies at title/abstract were located in the iteration which captured 95% of the title/abstract included studies.
- *The number of records screened that were excluded* (title/abstract TN). Reviews that have a large number of records that were included based on the title/abstract to be further reviewed at full text will likely have a higher rate of total number of records screened. Therefore, the number of excluded records screened was also recorded as this is the number of records that should be reduced to accurately report the reduction in screening burden.
- *The list of reference identification numbers (IDs) of the 5% of included records at title/abstract not yet identified* (title/abstract FN). This allowed for evaluation if any of these studies were on the list of final included studies in the systematic review (i.e., *final include*).

### Outcomes

The combined results from the 10 simulations per SR allowed for the calculation of the mean (SD) and median (range), when reporting results for a specific review, or median [interquartile range (IQR)] when reporting results across reviews for each outcome of interest:

1. The number and percent of records (at title/abstract) needed to screen to identify a true recall @ 95% (i.e., screening burden).
  Calculation: title/abstract TP + title/abstract TN (at a true recall @ 95%)
2. The number and percent of studies at title/abstract not yet identified at true recall @ 95% (title/abstract FN) among all studies that were included for further evaluation at full-text (title/abstract TP) at a true recall @ 100%.
  Calculation: [(title/abstract TP – title/abstract FN) / title/abstract TP]. As we are using a true recall @ 95%, this should approximate 5%.
3. The number and percent of final includes (i.e., those in the final list of included studies in the systematic review) among the title/abstract FN.
4. Number of hours saved, which was calculated using a modified screening approach, in which the AI reviewer would exclude all remaining records and a human reviewer would review these records. The number of hours saved was calculated by multiplying the expected time to review a record (i.e., one record per minute, based on Shemilt 2016 [11] and the experience of our own research groups) by the total number of records that did not need to be screened by one reviewer (i.e., the total number of records remaining once a true recall @ 95% was achieved). As this outcome is based on true recall, rather than estimated recall, the number of hours saved is an estimate as, in a prospective review, a review team would not know for certain if the estimated 95% was in fact 95% of the studies that would have been passed through to full-text screening, as not all references would have been screened.

### Deviations from the protocol

In the protocol, we stated that we would measure total cost savings as an outcome. However, the research team subsequently decided it would be of greater information and generalizability to knowledge synthesis researchers if we instead presented the number of hours saved. This would allow other researchers to calculate cost savings in different currencies at different salaries, as appropriate. Additionally, as the 95% modified screening approach resulted in a substantial number of records that did not need to be screened for some of the SRs, we performed an additional analysis to evaluate the difference in the relative screening burden when comparing how much of the total dataset was required to be screened to achieve a true recall @100% compared to a true recall @ 95%.

## Results

### Overview of SRs assessed

Ten SRs, consisting of 69,663 records, were used in this experiment. Four SRs included only RCTs, and the remaining SRs included both RCTs and observational studies. Using the review typology by Munn et al. (2018) [33], eight SRs were classified as effectiveness reviews [including both SR and network meta-analysis (NMAs)], and two SRs were effectiveness and etiology reviews. All SRs covered clinical areas and primarily evaluated the efficacy and safety of pharmacological, non-pharmacological (e.g., behavioural therapies), and surgical interventions. One SR each evaluated depression screening effectiveness, the use of e-cigarette for smoking cessation, and interventional/behavioural exposure to sugar sweetened beverages (SSBs) (Additional file 1). Reviews ranged in size from 2250 to 22,309 records to be assessed at title and abstract level, of which 3.0 to 39.2% (median: 16.2%) were included, based on the title/abstract, to be further reviewed at full text. A median of 0.6% (range 0.02 to 1.48%) of the total number of records were included in the final systematic reviews.

### Findings: reduction in screening burden

Across the set of 10 SRs evaluated, the median percentage of studies required to be screened to achieve a true recall @ 95% was 47.1% (IQR: 37.5 to 58.0%) (Table 2 and Additional file 3: Suppl. Table 1). Four SRs [i.e., non-small cell lung cancer, smoking cessation, prophylaxis for human immunodeficiency virus (HIV), SSBs] required at least 50% of records to be screened to achieve a true recall @ 95%. All of these reviews had more than 22% of the title/abstract records passed through for full-text screening. Among all reviews, when considering only the number of excluded records required to be screened to achieve true recall @ 95%, a median of 41.2% excluded records needed to be screened (IQR: 33.4 to 46.9%) (Additional file 3: Suppl. Table 1).

**Table 2:** Study results

Project	Project details ^**a**^	Iteration details	# of records needed to screen to achieve true recall @ 95% ^**b**^	Title/abstract includes not yet identified	Hours saved at title/abstract ^**b** c^	Final included studies missed
			Mean (%) [SD]; Median (range)			
Hot flashes	2569; 451 (17.6%); 38 (1.48%)	51 records; 17 or 18 iterations	892.5 (34.7%) [26.9]; 892.5 (867–918)	19.2 (4.3%) [2.04]; 19 (15–22)	27.9 [0.45]; 27.9 (27.5–28.4)	0
Opioid use disorder	16,282; 984 (6.0%); 71 (0.44%)	200 records; 23 or 23 iterations	4480 (27.5%) [103.3]; 4400 (4400–4600)	46.1 (4.7%) [3.38]; 48 (41–49)	196.7 [1.72]; 198.0 (194.7–198.0)	0
Meniere’s disease	2889; 332 (11.5%); 23 (0.80%)	57 records; 19–22 iterations	1168.5 (40.5%) [55.4]; 1140 (1083–1254)	15.0 (4.5%) [1.33]; 15.5 (12–16)	28.7 [0.92]; 29.2 (27.3–30.1)	0
Non-small cell lung cancer	3145; 795 (25.3%); 13 (0.40%)	62 records; 29 or 30 iterations	1829 (58.2%) [0.01]; 1829 (1798–1860)	33.7 (4.2%) [3.53]; 32.5 (29–39)	21.9 [0.54]; 21.9 (21.4–22.5)	0
Prophylaxis for influenza	8278; 395 (4.8%); 104 (1.26%)	165 records; 18 or 19 iterations	3019.5 (36.5%) [79.7]; 2970 (2970–3135)	18.8 (4.8%) [0.42]; 19 (18–19)	87.6 [1.33]; 88.5 (85.7–88.5)	0
Smoking cessation	2250; 881 (39.2%); 14 (0.62%)	45 records; 35 iterations	1575 (70.0%) [0]; 1575 (0)	39.9 (4.5%) [2.60]; 40 (34–44)	11.3 [0]; 11.3 (0)	0
Asthma/ Urticaria	3265; 482 (14.8%); 12 (0.36%)	65 records; 22 or 23 iterations	1488.5 (45.6%) [20.55]; 1495 (1430–1495)	22.5 (4.7%) [1.51]; 23 (20–24)	29.6 [0.34]; 29.5 (29.5–30.6)	0
Depression screening	4174; 126 (3.0%); 1 (0.02%)	83 records; 23–26 iterations	2025 (48.5%) [70]; 1992 (1909–2158)	5.8 (4.6%) [0.42]; 6 (5–6)	35.8 [1.17]; 36.4 (33.6–37.8)	0
Prophylaxis for HIV	4502; 1184 (26.4%); 46 (1.02%)	90 records; 30 iterations	2700 (60.0%) [0]; 2700 (0)	53.7 (4.5%) [1.49]; 53.5 (52–56)	30.0 [0]; 30.0 (0)	0
SSBs	22,309; 4993 (22.4%); 127 (0.57%)	200 records; 64 iterations	12,800 (57.4%) [0]; 12800 (0)	242.7 (4.9%) [2.06]; 243 (238–246)	158.5 [0]; 158.5 (0)	0

*HIV* Human immunodeficiency virus, *SD* Standard deviation, *SSB* Sugar sweetened beverage

^a^ Total number of records; Number of includes at title/abstract (% of all records); Number of included studies in the SR (% of all records)

^b^ Where there was no SD or range, this is identified by a 0

^c^ Hours saved at title/abstract = [(Total records - # of records needed to screen to identify 95% of includes)/60

**Fig. 2:**
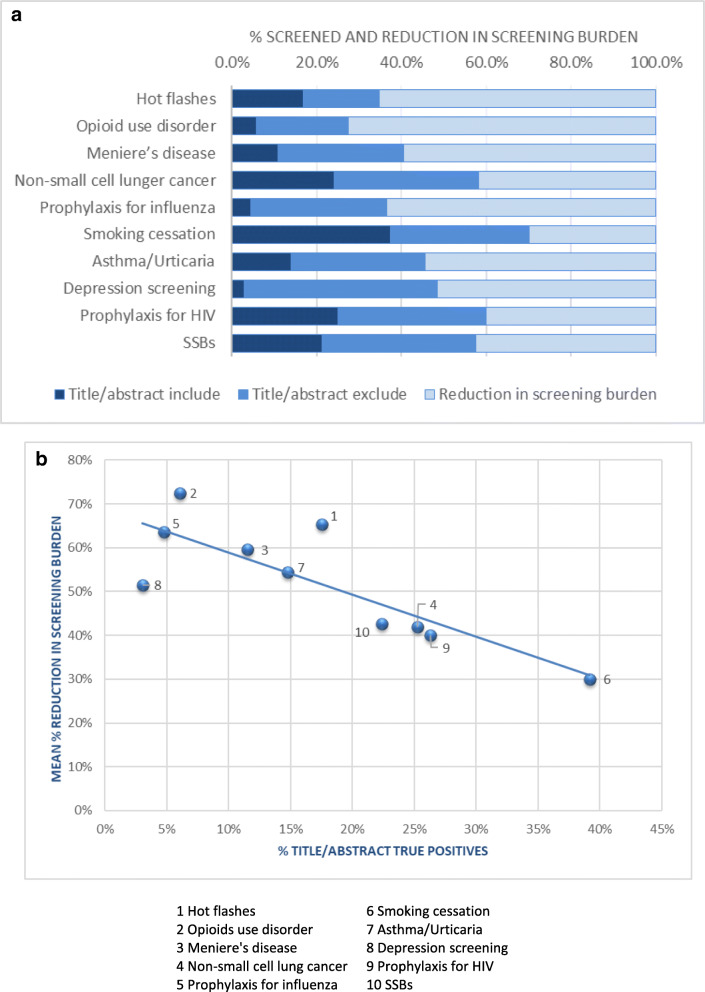
**a** Title/abstract includes and excludes and screening burden reduction. **b** – Relationship of mean % reduction in screening burden and % of title/abstract includes

Figure 2a presents the mean percentage of records that were included and excluded based on titles/abstracts, and the resulting reduction in the screening burden. The number of records that did not need to be screened (light blue portion of the bar) ranged from 30% (smoking cessation) to 72.5% (opioid use disorder). Figure 2b presents the relationship between the percentage of studies passed through to full-text screening and the mean percentage reduction in screening burden once true recall @ 95% was achieved. Typically, reviews with fewer studies passed through to full-text screening resulted in a larger reduction in the overall screening burden, as fewer excluded records would need to be screened to identify the studies requiring further review at full text.

There was little variation in the magnitude of screening burden within each of the 10 SRs among the 10 simulations. Three SRs achieve true recall @ 95% in the same number of iterations, while five SRs had a range of one iteration, and two SRs had a range of four iterations. It was common for the same references to be missed in each iteration. The difference between the total number of unique title/abstract included studies not yet identified (i.e., title/abstract FN that were listed in at least one of the ten simulations) and the largest number of title/abstract FN (i.e., the iteration with the largest number of title/abstract FNs) was 0 to 13 records [mean (SD): 5.3 records (4.03); median (IQR): 5 (2–8) records].

**Fig. 3:**
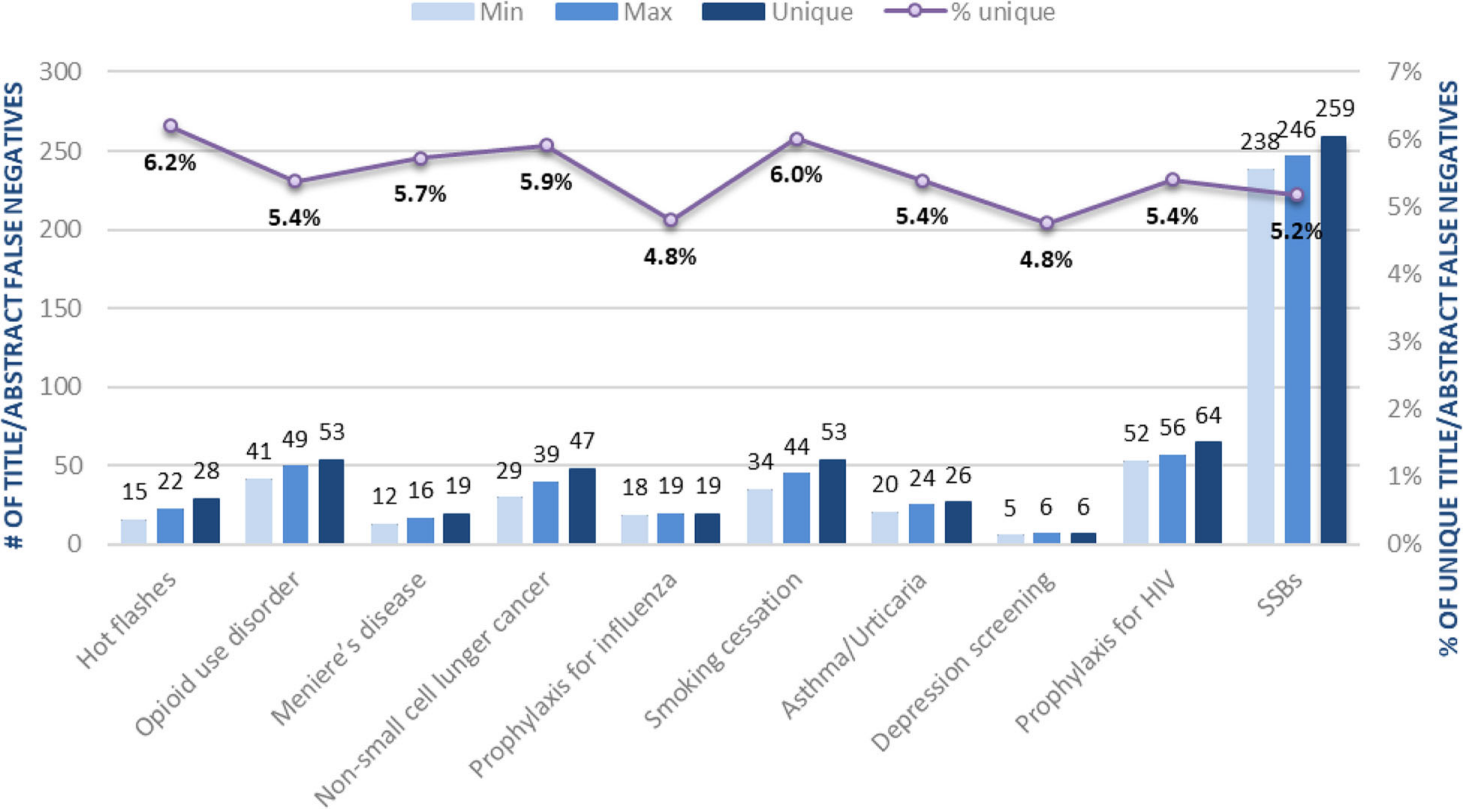
Title/abstract includes not yet identified (i.e., title/abstract false negatives)

Figure 3 presents the variation in the number of title/abstract included studies not yet identified (i.e., title/abstract FN) the simulation with the lowest number, highest number, and overall unique number of title/abstract FN. The lower the variation between simulations, the closer the minimum, maximum and number of unique studies. In these 10 reviews, 4.8 to 6.2% of the same records were not yet identified in the 10 simulations.

### Findings: amount of time saved

Overall, the mean title/abstract screening hours saved when using the true recall @ 95% modified screening approach (i.e., the AI reviewers would exclude all remaining references and one human review would be required to screen the remaining records) was 62.8 h (median: 29.8 h; IQR: 28.1 to 74.7 h). As would be expected, SRs with a larger number of records tended to result in more hours saved. SRs with fewer than 5000 records saved between 11.3 to 36 h. SRs with more than 5000 records (i.e., prophylaxis for influenza, opioid use disorder, and SSBs), saved totals of 88, 158 and 197 h (up to approximately 5 weeks of work time), respectively.

**Fig. 4:**
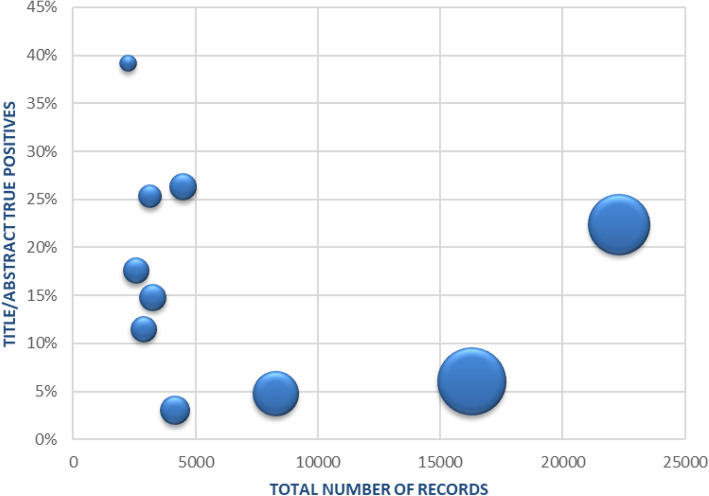
Mean hours saved in title/abstract screening using a true recall @ 95% modified approach

Figure 4 displays the mean hours saved per review from implementing the modified screening approach once a true recall @ 95% was achieved. The size of the bubbles represent the amount of hours saved. Reviews with fewer than 5000 records showed little variation in the total hours saved when the title/abstract true positive rate was between 10 and 30% (range 22 to 30 h, or approximately 1 day of work).

Using estimates from Shemilt et al. [11] of 4 min per person to retrieve a full text record and 5 min per person to screen a full text record, and assuming that full-text screening is done in duplicate, this would increase the total hours saved by not having to access and screen the 5% of title/abstract false negatives (Additional file 3: Suppl. Table 2). For example, in the review where AI was the least efficient in reducing the screening burden (i.e., smoking cessation), an average of 40 records did not need to be screened at title/abstract, a time savings of 11.3 h. However, adding the time to retrieve these articles (40 @ 4 min/record = 2.7 h) and the time for two reviewers to screen at full text (40 @ 5 min/record × 2 = 6.7 h), this results in an additional 9.4 h of time savings, nearly doubling the time savings. The Asthma/Urticaria review (which approximated the median for total records, % of includes at title/abstract, and time savings in hours) would result in a total time savings of 35.3 h (title/abstract screening: 30 h; retrieving full texts: 1.5 h; screening full texts: 3.8 h). The largest review, SSBs, would result in a total time savings of 215.1 h (title/abstract screening: 158.5 h; retrieving full texts: 16.2 h; screening full texts: 40.5 h). These numbers do not include any ordering fees for articles not accessible without a journal subscription, plus any additional time to resolve conflicts at full text (which has been estimated to take 5 min per conflict [11]).

**Fig. 5:**
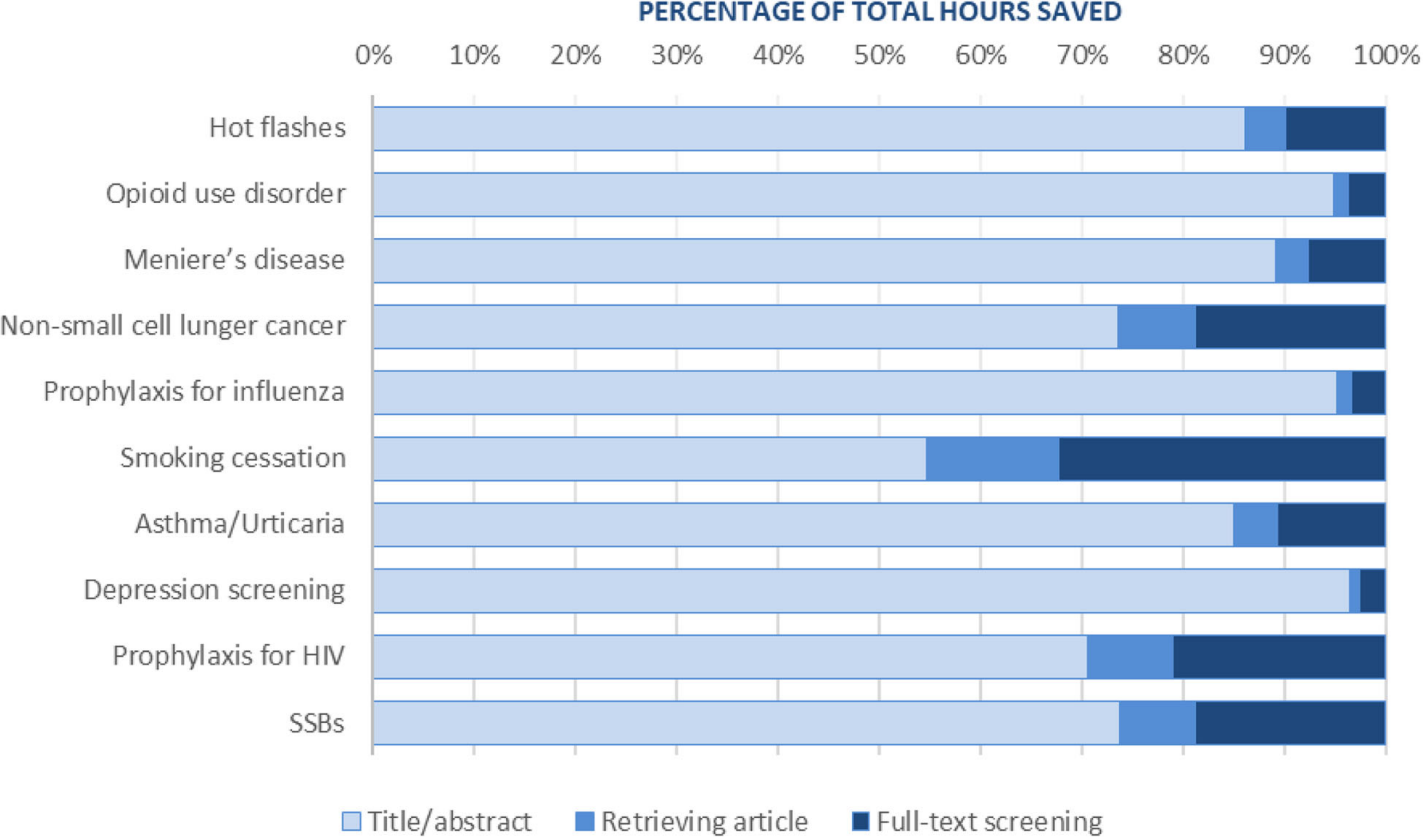
Estimated total time saved

Figure 5 shows that the extra time to retrieve the full text and perform full-text screening represents 4 to 45% of the estimated total time saved (median: 14%).

### Findings: performance (accuracy) of the prioritization algorithm

Across the 10 SRs studied, a median of 4.57% of the records were title/abstract FN (IQR: 18.9 to 44.6). Among the 100 iterations (10 iterations in 10 SRs), no final included studies were not yet identified at a true recall @ 95% (Table 2).

A post-hoc analysis was subsequently performed to evaluate the difference in the screening burden to achieve a true recall @ 100% compared to a true recall @ 95%. In measuring this, using the mean over three simulations, this resulted in a median difference in the number required to screen of 40.6% (IQR: 38.3 to 54.2%). It is important to note that the additional screening burden to identify the last 5% of the records included at title/abstract would not have identified any final included studies in the systematic reviews, as they were all identified in the true recall @ 95%.

**Fig. 6:**
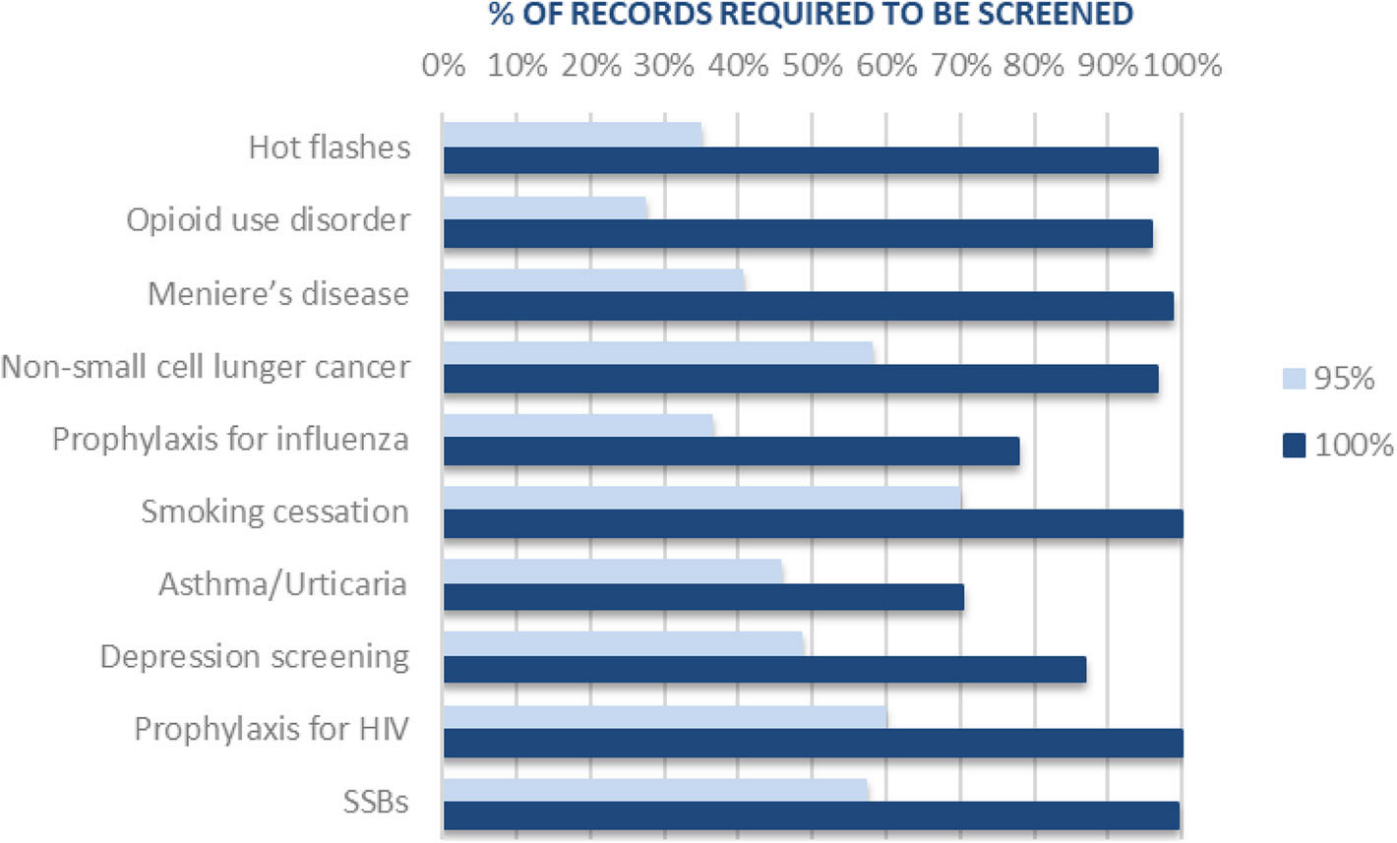
Screening burden to achieve true recall @ 95% and @ 100%

Figure 6 displays the reduction in screening burden over the 10 reviews at a true recall rate of 95 and 100%. Seven of the 10 reviews required over 90% of the records to be screened to achieve a true recall @ 100%. Two of these were the largest reviews (i.e., Opioids use disorder = 16,282 records, SSBs = 22,309 records).

## Discussion

The new prioritization tool in DistillerSR reduced the screening burden in these 10 SRs by 30.0 to 72.5% when using a true recall @ 95% modified screening approach. Smaller studies with a high inclusion rate will take longer to identify 95% of the title/abstract includes and resulted in poorer performance for the machine learning algorithm. Although some of the larger studies had high rates of title/abstract includes, due to the size of the dataset, the reduction in screening burden would still result in a large time and potentially lead to a subsequent cost savings. A recently published study evaluated the accuracy of screening prioritization of Abstrackr and EPPI-Reviewer [15]. Screening burden to identify all title/abstract includes for the de novo review was 85% or more for seven of the nine reviews for both Abstrackr (median: 93.8%, range: 71.1 to 99.0%) and EPPI-Reviewer (median: 91.3%, range: 39.9 to 97.9%). However, six of the nine included reviews had fewer than 1000 records, thereby not starting with a particularly large screening burden. Although not a direct comparison to our experiment, as different datasets were used, identifying 100% of the title/abstract includes using DistillerSR produced similar results (median: 96.6%, range: 70.3 to 100%). As there were no final includes missed with the true recall @ 95%, the extra screening burden to identify the last 5% of studies would not have changed the final results and conclusions, and may not be worth the additional efforts. Although this could be further evaluated, this suggests this last 5% of records where passed through for full-text screening due to either human error or a tendency toward over-inclusiveness while screening titles/abstract, and/or title/abstracts that were unclear, or records with no abstract which were included based on the title only. Other research teams are encouraged to use the information we have provided in order to build the evidence base.

There are several considerations to keep in mind when deciding to use prioritized screening in prospective reviews. It is important to have a clean (e.g., all duplicates removed) dataset, as any duplicates with conflicting decisions on whether to pass through for full-text screening or exclude based on the title/abstract would confuse the machine learning algorithm. Due to the retrospective nature of this experiment, this was not checked, as the assumption was made that this was performed when the SRs were originally conducted. Second, as the success of machine learning is dependent on the quality of the training set created by human reviewers, a precise training set (i.e., correctly designating title/abstract records) is required. A 2020 study by Wang et al. reported a 10.8% (95% confidence interval 7.4 to 14.1%) error rate (i.e., incorrectly included or incorrectly excluded at title/abstract screening) among 139,467 citations that underwent 329,332 inclusion and exclusion decisions [3]. Although incorrectly excluding a record at title and abstract level is more concerning, as this record is no longer considered for inclusion, incorrectly passing a record at title and abstract for further review at full text increases screening burden at full text, in addition to the time and costs associated with retrieving the full-text articles. It is therefore important to ensure that a pilot test is first performed with conflicts resolved, that all reviewers are confident in their assessments (i.e., do not include because of uncertainty of reviewer rather than uncertainty of relevance), and that conflict resolution is performed throughout screening. Review team may also choose to set up reviewer compatibility (if the software permits), where junior reviewers are unable to screen the same references. This may decrease the number of records that are incorrectly included due to uncertainty.

### Limitations

There were some limitations in the conducted study. First, screening at the title and abstract level in the set of systematic reviews we studied was performed using the liberal accelerated method [34], which requires two reviewers to exclude a reference, but requires only one reviewer to include a reference to be further evaluated at full text. Further, any conflicts resulting from the first reviewer excluding and the second reviewer including were not resolved. This presents two limitations: (i) there may be a tendency to be over-inclusive while screening titles/abstracts as only one reviewer is required to pass the reference through for further full-text screening; and (ii) by using retrospective responses, the machine-learning algorithm is not able to distinguish between records that were excluded by the first reviewer and later included by the second reviewer. These records may be less likely to be true includes. As a training set with high accuracy (i.e., true title/abstract includes and true excludes) will result in fewer excluded references required to be screened to achieve true recall @ 95%, over-inclusiveness of records likely resulted in poorer performance of the AI tool. Second, this experiment was only conducted using DistillerSR, which might not be generalizable to all prioritization algorithms and related software.

### Implications for future research

In this pilot experiment evaluating the AI simulation tool in DistillerSR, we selected 10 reviews which included a variety of review types (e.g., NMAs, SRs of RCTs, SRs including observational studies), sizes (ranging from 2250 to 22,309 records), and inclusion rates (ranging from 3.0 to 39.2% at title/abstract screening). We encourage other review teams to use the guidance provided in Additional file 2 to evaluate the AI simulation tool on their own projects. For review teams who do not have access to DistillerSR or who do not have the resources to run these experiments, the authorship team of this study plans on increasing the sample size of this experiment by asking other review teams to provide their databases so this experiment can be run. We plan to establish a website for this work that will allow for the provision of updated findings in an ongoing fashion. Offers to contribute to this initiative will be shared with other teams in the future through email, social media and other forms of communication.

In the context of rapid reviews, a form of knowledge synthesis that accelerates the process of conducting a traditional systematic review through streamlining or omitting a variety of methods to produce evidence in a timely and resource-efficient manner [27–30], identification of fewer than 95% of the title/abstract true positives may be acceptable. A survey of stakeholders (e.g., policy-makers, healthcare providers) reported that the median acceptable incremental risk of getting an incorrect answer from a rapid review is 10% (interquartile range of 5–15%) [35]. A missed study (or studies) does not imply there will be an incorrect answer, depending on the study (ies), as missed studies may not change the overall conclusion appreciably in terms of either direction or magnitude of effects studied. Therefore, the decision to stop screening or change the method of screening (e.g., from dual-independent to single screener) once another percentage of studies passed through for full-text review have been identified (e.g., 75, 85%) may be further evaluated.

As true recall can only be calculated once all records are screened, estimated recall might differ depending on how quickly relevant records (at title/abstract) are identified. For example, an estimated recall @ 95% may only be accounting for 91% of the included records if all were screened. Therefore, a review team might not be comfortable to implement a modified or stop screening approach when an estimated recall of 95% is first achieved. They may consider screening an additional set of records (e.g., two to four more iterations) to confirm no new title/abstract records are passed through for full-text screening. Estimated recall rates may be further evaluated to determine the difference between estimated and true recall rates and how many more records should be screened once a certain estimated recall threshold has been achieved.

Prospective studies using the prioritization tool should be performed that report transparent and repeatable methods. These steps might change the process by which review teams currently conduct their systematic reviews. For example, although not an option when using the AI simulation on a previously completed review, in a prospective review using prioritization, review teams are encouraged to use dual-independent screening at the title and abstract level, with conflicts resolved throughout the screening process (e.g., after every 10% of references screened, at the end of each day) to minimize over-inclusiveness and maximize the performance of the AI prioritization tool. Review teams are also encouraged to use the *Check for Error* audit throughout screening to ensure that no references are incorrectly excluded, although this should be rare when performing dual-independent screening. Prospective studies may contribute to a set of best practices for using prioritized screening, and may also help to inform a future reporting checklist for protocols and manuscripts for these types of experiments or for reviews (e.g., systematic, rapid) using AI.

## Conclusion

Our findings from this study suggest that the prioritization tool in DistillerSR can reduce screening burden. Even for reviews where the tool performed less efficiently, the time savings were still appreciable. Modified or stop screening approaches once a true recall @ 95% has been achieved appears to be a valid method for rapid reviews, and perhaps systematic reviews, as it did not miss any of the final includes studies in the systematic review.

## Supplementary information

**Additional file 1.** Systematic review details [36–44].

**Additional file 2.** Steps for Testing Prioritization in DistillerSR through AI Simulation.

**Additional file 3. Supplementary Tables: Table 1.** Records required to screen to achieved true recall @ 95%. **Table 2.** Total hours saved.

## Data Availability

The datasets used and/or analysed during the current study are available from the corresponding author on reasonable request.

## Abbreviations

AI: Artificial intelligence
HIV: Human immunodeficiency viruses
IQR: Interquartile range
NMA: Network meta-analysis
RCT: Randomized controlled trial
SD: Standard deviation
SR: Systematic review
SSBs: Sugar sweetened beverages
