# Supplementary material for: An evaluation of DistillerSR’s machine learning-based prioritization tool for title/abstract screening – impact on reviewer-relevant outcomes
Source: BMC Med Res Methodol. 2020 Oct 15;20:256. doi: 10.1186/s12874-020-01129-1 (PMC7559198; doi:10.1186/s12874-020-01129-1)
Supplement: Supplementary file 1 — Additional file 1. Systematic review details [36–44]. [file 12874_2020_1129_MOESM1_ESM.docx]

# Additional file 1. Systematic review details

| **Systematic review [Protocol reference]** | **Review short name** | **Review type**$\boldsymbol{\dagger}$ | **Intervention type** | **Study designs included** | **Number of studies at** | | |
| --- | --- | --- | --- | --- | --- | --- | --- |
|  |  |  |  |  | **Ti/Ab** | **Full-text** | **Final⁑** |
| Interventions for hot flashes [36] | Hot flashes | Effectiveness review (NMA) | Pharmacological and behavioural therapies | RCTs only | 2569 | 451  (17.6%) | 38  (1.48%) |
| Interventions for opioid use disorder [37] | Opioid use disorder | Effectiveness review (NMA) | Psychosocial therapies | RCTs only | 16282 | 984  (6.0%) | 71  (0.44%) |
| Interventions for Meniere’s disease [38] | Meniere’s disease | Effectiveness (NMA) | Pharmacological therapies, surgical interventions | RCTs only | 2889 | 332  (11.5%) | 23  (0.80%) |
| ALK inhibitors for non-small cell lung cancer [CRD42017077046] [39] | Non-small cell lung cancer | Effectiveness review (NMA) | Pharmacological therapies | RCTs & observational | 3145 | 795  (25.3%) | 13  (0.40%) |
| Influenza vaccination during pregnancy [<https://osf.io/xey2k/>] | Prophylaxis for influenza | Effectiveness review | Pharmacological intervention | RCTs & observational | 8278 | 395  (4.8%) | 104 (1.26%) |
| E-cigarettes for smoking cessation [40] | Smoking cessation | Effectiveness review | E-cigarette | RCTs & observational | 2250 | 881  (39.2%) | 14  (0.62%) |
| Omalizumab for atopic asthma and chronic idiopathic urticaria  [CRD42018082211] [41] | Asthma/ Urticaria | Effectiveness and safety review | Pharmacological intervention | RCTs & observational | 3265 | 482  (14.8%) | 12  (0.36%) |
| Adult depression screening [42] | Depression screening | Effectiveness review | Screening intervention | RCTs only | 4174 | 126  (3.0%) | 1  (0.02%) |
| Antiretroviral drugs and implementation strategies for HIV pre-exposure prophylaxis [CRD42017073014] | Prophylaxis for HIV | Effectiveness & Etiology review | Pharmacological and behavioural therapies | RCTs & observational | 4502 | 1184  (26.3%) | 46  (1.02%) |
| Sugar sweetened beverages and adverse health outcomes [43,44] ‡ | SSBs | Effectiveness & Etiology review | Interventional/ behavioural and exposure to SSBs | RCTs & observational | 22309 | 4993  (22.4%) | 127 (0.57%) |

NMA: Network Meta-analysis; RCT: Randomized Controlled Trial; SR: Systematic Review

† Based on typology classifications from Munn 2018 [35]

‡ Protocol published as two separate reviews, with references screened in one project silo.

⁑ The final included studies does not always reflect the study designs that were included in the protocol, as decisions may have been made during the process of the review. However, the study designs reflect those that were included in the search strategy and screened at title/abstract.

**Intervention for hot flashes** [36]

- *Review Question(s)/Objectives*: In breast cancer and prostate cancer survivors, what are the relative benefits of non-hormonal therapies on (1) frequency and severity of hot flashes? (2) quality of life? (3) quality of life related to depression and sleep quality?
- *Review type*: Effectiveness review (network meta-analysis)
- *Participants*: Patients diagnosed with breast cancer or prostate cancer and who are experiencing hot flashes.
- *Interventions and Comparators*: Non-hormonal pharmacologic, behavioral/physical, and natural health products.
- *Primary outcome*: Changes in the intensity and frequency of hot flashes, with or without night sweats.
- *Study designs included*: RCTs and cross-over trials.

**Interventions for opioid use disorder** [37]

- *Review Question(s)/Objectives*: To compare the relative benefits of different psychosocial therapies among people with opioid use disorder receiving opioid agonist therapy (OAT).
- *Review type*: Effectiveness review (network meta-analysis)
- *Participants*: Individuals with problematic opioid use receiving treatment pharmacological (opioid agonist treatment, OAT) and psychosocial interventions for opioid use.
- *Interventions:* Psychosocial interventions (delivered with OAT). Studies evaluating the benefits of psychosocial interventions alone will be excluded.
- *Comparators:* Studies using control groups of either OAT alone or ‘standard medical management’.
- *Co-primary outcomes*: Treatment retention and opioid use (including abstinence from opioids and opioid use based on urinalysis results).
- *Study designs included*: RCTs

**Interventions for Meniere’s disease (MD)** [38]

- *Review Questions/Objectives*: Primary objective was to assess the relative effects of available pharmacologic therapies in patients with MD on vertigo and other key patient outcomes. The secondary objective was to assess the effects of surgical interventions in the MD population on vertigo and other key patient outcomes.
- *Review type*: Effectiveness review (network meta-analysis)
- *Participants*: Adult patients with MD per established criteria (i.e., American Academy of Otolaryngology-Head and Neck Surgery (AAOHNS)) receiving pharmacologic or surgical interventions for their condition. Patients were required to be unresponsive to a prior intervention in order to be treated with the intervention(s) of interest.
- *Interventions and comparators*: Systemic pharmaceuticals, intra-tympanic pharmaceuticals, surgical interventions (details for each type available in protocol).
- *Outcomes*: frequency, severity, type, and control of vertigo; occurrence and intensity of tinnitus; changes in hearing; quality of life; perception of aural fullness; and harms.
- *Study designs included*: RCTs and quasi-RCTs

**ALK inhibitors for non-small cell lung cancer (NSLC)** [Prospero: CRD42017077046] [39]

- *Review Question(s)/Objectives*: To identify all RCTs involving the use of any ALK inhibitor to treat ROS1 or ALK-positive NSCLC.
- *Review type*: Effectiveness systematic review
- *Participants*: Treatment-naïve or experienced participants with phase III or IV ALK-positive and/or ROS1-positive NSLC
- *Intervention:* ALK inhibitors (e.g, crizotinib, ceritinib, alectinib, brigatinib, loratinib, ensartinib, and entrectinib)
- *Comparators:* Placebo, chemotherapy, radiotherapy, another ALK inhibitor, or the same ALK inhibitor at a different dose.
- *Outcomes*: Treatment-related death; Overall survival; Progression-free survival; and SAEs as reported by the study authors.
- *Study designs included*: RCTs, non-randomized studies (observational) with and without controls (prospective and retrospective cohort studies, historically controlled trials, case-series reports with at least 50 participants†, case-control), abstracts reporting on one of the eligible study designs will be examined for reporting of the primary outcome †If insufficient data are located, a less stringent cutoff of 10 patients may be considered.

**Influenza vaccination during pregnancy** [<https://osf.io/xey2k/>]

- *Review Questions/Objectives:* To assess the safety and effectiveness of influenza vaccines for women and their newborns if received at any time during their pregnancy. Specific research questions: 1. Are influenza vaccines safe for women and their newborns if received at any time during pregnancy? 2. Are influenza vaccines effective for preventing influenza and its complications for women and their newborns if received at any time during pregnancy?
- Review type: Effectiveness systematic review
- *Participants*: Women at any stage in their pregnancy and infants less than 6 months of age.
- *Interventions:* Trivalent or quadrivalent inactivated influenza vaccine; cell-based and Recombinant Influenza Vaccine (RIV) indicated for adults and not contraindicated in pregnancy; and/or monovalent pandemic influenza vaccines (e.g., H1N1 vaccines).
- *Comparators*: No influenza vaccine, placebo, or active comparators (e.g., pneumococcal and meningococcal vaccine).
- *Outcomes*: Maternal: vaccine effectiveness against lab-confirmed influenza infection, lab-confirmed influenza hospitalization, and influenza-associated outcomes (i.e., those that are not necessarily lab-confirmed); stillbirths; spontaneous abortion; serious non-obstetric adverse events (AEs). Infant: vaccine effectiveness against lab-confirmed influenza infection, lab-confirmed influenza hospitalization, and influenza-associated outcomes, i.e. indirect protection; preterm birth; small-for-gestational-age birth; low birthweight birth; congenital anomalies; early neonatal death (within 7 days of birth); death within 6 months.
- *Study designs*: Randomized clinical trial, quasi-randomized trial; non-randomized, comparative observational studies; registry of adverse events of interest

**E-cigarettes for smoking cessation** [40]

- *Review Questions/Objectives:* To evaluate the benefit and harms of e-cigarettes to promote cessation of tobacco smoking among adults. Specific research questions: 1. What are the benefits and harms of electronic cigarettes for tobacco smoking cessation in adults? 2. What is the comparative effectiveness (benefits and harms) of electronic cigarettes for tobacco smoking cessation in adults?
- Review type: Effectiveness systematic review
- *Participants*: Adults (≥ 18 years) who are current tobacco smokers.
- *Interventions:* Nicotine or non-nicotine containing e-cigarettes, Nicotine or non-nicotine containing e-cigarettes combined with other smoking cessation treatment (behaviour and/or pharmacological)
- *Comparators*: Question 1: Non-nicotine containing e-cigarettes (i.e. placebo e-cigarettes), No intervention, Usual/standard care, Waitlist, Minimal intervention. Question 2: Alternative nicotine containing e-cigarettes (e.g. different generation e-cigarette or e-cigarette containing a different dose of nicotine), Non-nicotine containing e-cigarettes, Other smoking cessation aids (e.g. nicotine replacement therapy).
- *Outcomes*: Tobacco use abstinence (as defined in the study); Reduction in tobacco smoking frequency/quantity; Relapse (Question 2 only); Quality of life (using validated scales); Adverse events (as defined in a given review); Possible adverse outcomes including weight gain, changes in emotional state (e.g. increases in anxiety, changes in mood, irritability), and loss of social group.
- *Study designs*: For benefits: Randomized controlled trials. For harms: Randomized controlled trials, Non-randomized controlled trials, Comparative observational study designs (e.g. prospective and retrospective cohort studies, case-control studies).

**Omalizumab for atopic asthma and chronic idiopathic urticaria** [Prospero: CRD42018082211] [41]

- *Review Question(s)/Objectives*: To investigate whether prolonged treatment with omalizumab influences the development or progression of solid epithelial cancer in patients (≥12 years of age) with atopic asthma or chronic idiopathic urticaria.
- *Review type*: Effectiveness and safety systematic review
- *Participants*: Adults and adolescents (≥12 years) with chronic idiopathic/spontaneous urticaria (CIU/CSU) or moderate‐to‐severe persistent allergic asthma who were treated with long‐term (continuous) omalizumab.
- *Intervention:* Interventional studies were eligible if the final dose of omalizumab was administered at 48 weeks or longer and observational studies were eligible if participants were treated with omalizumab for a minimum of 40 weeks.
- *Comparators:* Placebo, standard of care, sodium cromoglycate and no treatment.
- *Outcomes*: The number of study participants diagnosed with study‐emergent epithelial cancer (primary outcome), the number of participants diagnosed with study‐emergent epithelial cancers by organ system and histological type, as well as the number of participants whose current solid epithelial cancer progressed during the study treatment period (secondary outcomes).
- *Study designs included*: RCTs, quasi-randomized trials, controlled clinical trials, and observational studies (e.g., cohort, case-control).

**Adult depression screening** [42]

- *Review Question(s)/Objectives*: What are the benefits and harms of screening versus no screening for depression in the general adult population in primary care or other non-mental health clinic settings?
- *Review type*: Effectiveness systematic review
- *Participants*: Patients who are 18 years and older
- *Intervention:* Interventions that use a single question, small sets of questions, or a screening questionnaire (validated or non-validated) with a pre-defined cut-off score to identify patients who may have depression, but who have not reported their symptoms to healthcare providers or who have otherwise not been identified as possibly depressed by healthcare providers.
- *Comparators:* No depression screening. Patients in comparator trial arms may be administered depression symptom questionnaires for the purpose of baseline or outcome assessments as long as scores are not provided to the patients or healthcare providers.
- *Outcomes*: Symptoms of depression (continuous or dichotomous) or diagnosis of MDD (using a validated diagnostic interview); Health-related Quality of life; Day-to-day functionality; Lost time at work/school; Impact on lifestyle behaviour (alcohol abuse, smoking, drugs, gambling, etc.); Suicidality (suicide ideation, attempt or completion); False positive result (positive screen in absence of depressive disorder), overdiagnosis, or overtreatment; Labeling/stigma; Harms of treatment
- *Study designs included*: RCTs including cluster randomized trials. To be considered an included RCT: (i) the patient population must be clearly defined and participants randomized prior to administering the screening test; (ii) patients who are known to have a current episode of depression or are already being treated for depression close to the time of eligibility assessment are excluded, as screening is intended to identify undetected cases and those who are known to have depression would not be screened in actual clinical practice; and (iii) similar depression management and treatment resources must be provided to patients in the screening arm of the trial and patients in the non-screening arm of the trial who are identified as depressed via other methods (e.g., unaided clinician diagnosis, patient report).

**Antiretroviral drugs and implementation strategies for HIV pre-exposure prophylaxis** [Prospero: CRD42017073014]

- *Review Question(s)/Objectives*: What are the effects of antiretroviral drugs and implementation strategies for PrEP on HIV acquisition, adverse events, adherence rates and drug resistance in various HIV-negative populations? What are the factors that enable or impede the implementation of PreP?
- *Review type:* Effectiveness & Etiology systematic review
- *Participants:* HIV-negative individuals of any age, in the following high-risk populations: men who have sex with men (MSM), people who inject drugs (PWID), serodiscordant couples, sexually active young adults (15-24 years old, include if >80% of the participants fall into this category), sex workers, transgender individuals, and sexually active pregnant women.
- *Interventions:* Studies prescribing ARV drugs for PrEP [e.g., tenofovir, tenofovir/emtricitabine, rilpivirine, or dapivirine at any dose), formulation (tablet, topical vaginal/ anal gel, long-lasting injection, or intravaginal ring), and duration (intermittent or lifetime)]. ARV drug regimens may be used in combination with some form of implementation strategies [e.g., sexual health education, text messaging, personalized cognitive counseling, a peer navigator support group, mobile PrEP clinics, task-shifting (from physicians to public health nurses), mental health or psychosocial support services].
- *Comparators:* The presence of a comparator arm is not a requirement for inclusion. Studies with or without comparator arms will be included.
- *Outcomes:* Incidence of HIV infection; Adherence to PrEP; Incidence and frequency of adverse events and complications, such as impaired kidney function and decrease in bone mineral density; ARV drug resistance; Frequency of condom use, partner numbers, relative risk of bacterial sexually transmitted infections (e.g., syphilis, gonorrhea, chlamydia)
- *Study designs:* Experimental studies (e.g. RCTs, quasi-RCTs), Quasi-experimental studies (interrupted time series with at least 3 data points before and three data points after intervention, controlled before and after studies with at least 2 intervention and 2 control sites), and Prospective cohort studies.

**Sugar sweetened beverages (SSB) and adverse health outcomes** [43,44]

- *Review Question(s)/Objectives*: In adults/children, does the consumption of SSBs cause adverse health outcomes? If so, what potential moderating factors affect the causal association between SSB consumption and outcomes?
- *Review type*: Etiology systematic review
- *Participants*:
  - *Adult*: General adult human population or healthy adult humans (18 years or older).
  - *Children*: General human paediatric population or healthy human children aged 2–17 years.
- *Intervention/Exposure*: SSB consumption, taking a broad perspective. In addition to direct consumption studies, we would consider interventions that influence consumption, such as those addressing the level of access to SSBs (e.g. university/college policy/school policy) and educational interventions addressing consumption as relevant.
- *Comparators*: SSB consumption compared with consumption of non-SSB drink (e.g. 100% fruit juice, artificially sweetened beverage, water); Higher level of SSB consumption versus lower level of SSB consumption for the same drink type (e.g. carbonated cola beverages); Comparisons among different categories of SSBs (e.g. soft drinks compared with fruit drinks consumed in similar amounts; One level of access to SSB compared with another level of access (e.g. university/college policy on beverages in vending machines); Educational intervention to specifically promote lower or no SSB consumption compared with no educational intervention/regular curriculum coverage/general health-focused intervention; Non-specific or multi-faceted educational, behavioural, or policy dietary intervention (may include component of SSB consumption) compared with no intervention; Other comparisons involving interventions that address our research question (interventions assessed on a case-by-case basis, as encountered in the literature).
- *Outcomes*:
  - *Adults*: Endpoints important for decision-making: Adverse cardiovascular (including cerebrovascular) events; Cancer (excluding basal cell and squamous cell carcinoma); Chronic kidney disease; Mortality; Overweight/obesity; Type 2 diabetes; Dental caries; Quality of life (generic, validated tools only); Gout. Surrogate outcomes: Pre-diabetes; Metabolic syndrome; Change in cardiovascular disease (CVD) risk; Progression of obesity; Dyslipidemia; Hypertension.
  - *Children*: Endpoints important for decision-making: Academic achievement; Dyslipidemia; Fractures; Hypertension; Overweight/obesity/change in weight; Prediabetes/type 2 diabetes; Dental caries; Quality of life (generic, validated tools only). Surrogate outcome: Bone mineral density (BMD).
- *Study designs*: Randomized controlled trials, including cluster RCTs, controlled (non-randomized) clinical trials or cluster trials, interrupted time series studies with at least three data points before and after the intervention, controlled before-after studies, prospective and retrospective comparative cohort studies, and case-control or nested case-control studies.
