# Supplementary material for: An evaluation of DistillerSR’s machine learning-based prioritization tool for title/abstract screening – impact on reviewer-relevant outcomes
Source: BMC Med Res Methodol. 2020 Oct 15;20:256. doi: 10.1186/s12874-020-01129-1 (PMC7559198; doi:10.1186/s12874-020-01129-1)

# Additional file 2. Steps for Testing Prioritization in DistillerSR through AI Simulation

For research teams interested in performing these experiments with their own systematic review databases, the series of steps has been provided.

## Run the AI Simulation tool.

The AI simulation tool can be run on completed projects or on projects that are underway, as the simulation tool will not affect any of the data in the project. After the simulation tool randomizes the reference order, the tool then provides a projection on how many included studies at title/abstract have been found (Y axis) related to the number of excludes examined (X axis) (**Figure 1**). This is achieved using a random set of references as part of the initial training set and building on that information (as what would be done in prospective screening). The objective of using a prioritization software is to identify the majority of the title/abstract true positives while only requiring screening of a minority of the excluded studies. The blue line at 45 degrees represents the predicted rate at which title/abstract true positives would be identified if screening using random ordering of citations is used (i.e., screening using no machine-learning), as these studies would be expected to be scattered throughout the full set of excluded studies.

1. Under the *References* menu item, select *Distiller AI Toolkit* then click on the AI Simulation tab (default tab is AI Test).
2. With Level 1 select as the “Highest Screening Level”, click on the *Run simulation* button. This will run the simulation and produce a chart (**Figure 1**). In the example shown, 95% of the title/abstract true positives (the target for the planned experiment) were identified by the time 32% of the excluded studies were reviewed using prioritized screening.


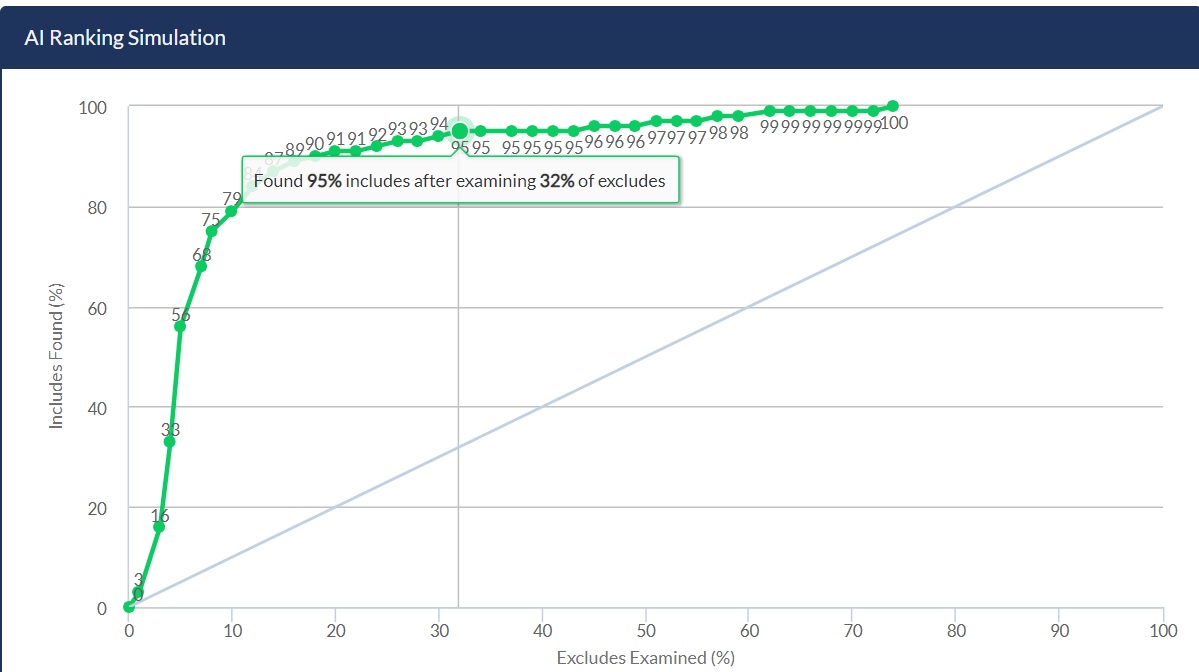


Figure 3 - AI Ranking Simulation chart

Each point on the chart represents an iteration (i.e., a set of records in the training set which are used to re-rank the remaining records). Each iteration is triggered based on a pre-specified number of records, 2% of the total number of records in the dataset, with a minimum of 25 records and a maximum of 200 records per iteration. If the radio toggle (on the main AI Simulation page) has been set to *Display Additional Information* (default is Yes), tabular information for each iteration is displayed below the simulation chart. These data present the number and percent of included citations (at title/abstract) found, the number and percent of excludes examined, the total number and percent of references examined, and a ratio of the excludes per includes (excludes divided by includes) per iteration. A histogram also displays the likelihood of inclusion at title/abstract (i.e., likelihood of title/abstract true positives) of the remaining, unscreened references (**Figure 2**).


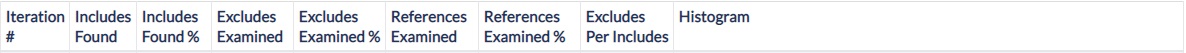

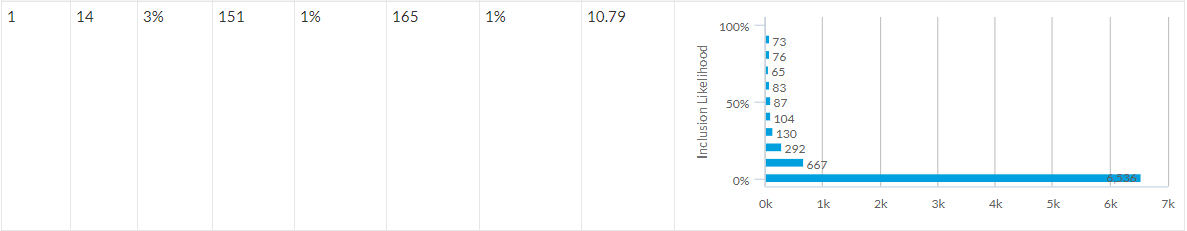


Figure 4 - Iteration #1 with inclusion and exclusion details

## Verified missed included studies at title/abstract

To verify if any of the final set of included studies (i.e., studies that were included in the review) were in the 5% of title/abstract false negatives, a listing of reference identification numbers (RefIDs) for citations included based on title/abstract not yet identified by the tool are displayed once 95% of the included studies at title/abstract are found (**Figure 3**). These RefIDs can be compared to the list of final included studies. The *Datarama* screen can be opened by clicking anywhere on these RefIDs to see the RefID, Title, and Abstract information. The histogram shown indicates that the likelihood of finding a record that would be passed through to full-text screening out of the remaining records is low (1 record at 10% likelihood of inclusion and 5307 records at 0% likelihood of inclusion).


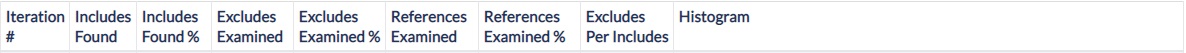

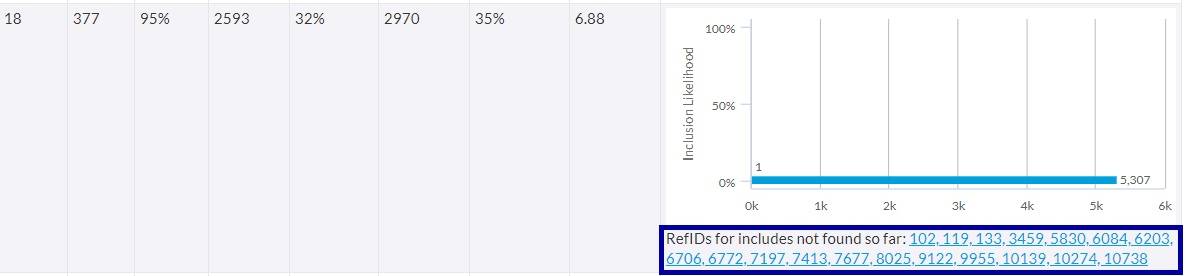

Supplement: Supplementary file 2 — Additional file 2. Steps for Testing Prioritization in DistillerSR through AI Simulation. [file 12874_2020_1129_MOESM2_ESM.docx]
