# Supplementary material for: An evaluation of DistillerSR’s machine learning-based prioritization tool for title/abstract screening – impact on reviewer-relevant outcomes
Source: BMC Med Res Methodol. 2020 Oct 15;20:256. doi: 10.1186/s12874-020-01129-1 (PMC7559198; doi:10.1186/s12874-020-01129-1)
Supplement: Supplementary file 3 — Additional file 3. Supplementary Tables: Table 1. Records required to screen to achieved true recall @ 95%. Table 2. Total hours saved. [file 12874_2020_1129_MOESM3_ESM.docx]

# Additional file 3. Supplementary Tables

Table 1. Records required to screen to achieved true recall @ 95%

Table 2. Total hours saved

## Supplementary Table 1. Records required to screen to achieved true recall @ 95%

Table 1 presents the mean and median total number of records and total number of excluded records that were screened to achieve true recall @ 95%. The goal of the prioritization tool is to minimize the number of excluded records that need to be screened.

| **Systematic Review** | **Total % (n/N) of records included at title/abstract** | **Mean after 10 simulations** | | **Median after 10 simulations** | |
| --- | --- | --- | --- | --- | --- |
|  |  | **Total % (n/N) of records screened** | **Total % (n/N) of excluded records screened** | **Total % (n/N) of records screened** | **Total % (n/N) of excluded records screened** |
| Hot flashes | 17.6%  (38/451) | 34.7%  (893/2569) | 21.8%  (461/2118) | 34.7%  (893/2569) | 21.7%  (460/2118) |
| Opioid use disorder | 6.0%  (984/16282) | 27.5%  (4480/16282) | 23.2%  (3542/15298) | 27.0%  (4400/2569) | 22.7%  (3465/15298) |
| Meniere’s disease | 11.5%  (332/2889) | 40.4%  (1169/2889) | 33.3%  (852/2557) | 39.5%  (1140/2889) | 32.2%  (824/2557) |
| Non-small cell lung cancer | 25.3%  (795/3145) | 58.2%  (1829/3145) | 45.4%  (1068/2350) | 58.2%  (1829/3145) | 45.4%  (1068/2350) |
| Prophylaxis for influenza | 4.8%  (395/8278) | 36.5%  (3020/8278) | 33.5%  (2643/7883) | 35.9%  (2970/8278) | 32.9%  (5308/7883) |
| Smoking cessation | 39.2%  (881/2250) | 70.0%  (1575/2250) | 53.6%  (734/1369) | 70.0%  (1575/2250) | 53.6%  (734/1369) |
| Asthma/Urticaria | 14.8%  (482/3265) | 45.6%  (1489/3265) | 37.0%  (1029/2783) | 45.8%  (1495/3265) | 37.2%  (1036/2783) |
| Depression screening | 3.0%  (126/4174) | 48.5%  (2025/4174) | 47.1%  (1905/4048) | 47.7%  (1992/4174) | 46.2%  (2182/4048) |
| Prophylaxis for HIV | 26.3%  (1184/4502) | 60.0%  (2700/4502) | 47.3%  (1570/3318) | 60.0%  (2700/4502) | 47.3%  (1570/3318) |
| SSBs | 22.4%  (4993/22309) | 57.4%  (12800/22309) | 46.5%  (8050/17316) | 57.4%  (12800/22309) | 46.5%  (8050/17316) |

HIV: Human Immunodeficiency Viruses; SSBs: Sugar sweetened beverages

## Supplementary Table 2. Total hours saved

Table 3 presents the hours saved for title/abstract screening (all records that did not need to be screened by one reviewer) and includes the time saved by not having to retrieve articles and screen at full text among the approximately 5% of the title/abstract false negatives. For some reviews, this extra time beyond title/abstract screening was small (e.g., depression screening = 1.4 hours), but for larger projects (i.e., SSBs) this resulted in an additional time savings of over 56 hours.

| **Systematic Review** | **Number of included title/abstract not yet identified** | **Time savings (in hours)** | | | |
| --- | --- | --- | --- | --- | --- |
|  |  | **Total hours saved** | **Title/abstract screening** | **Retrieving articles** † | **Full-text screening** ‡ |
| Hot flashes | 19 | 32.4 | 27.9 | 1.3 | 3.2 |
| Opioid use disorder | 46 | 207.5 | 196.7 | 3.1 | 7.7 |
| Meniere’s disease | 15 | 32.2 | 28.7 | 1.0 | 2.5 |
| Non-small cell lung cancer | 34 | 29.8 | 21.9 | 2.2 | 5.6 |
| Prophylaxis for influenza | 19 | 92.0 | 87.6 | 1.3 | 3.1 |
| Smoking cessation | 40 | 20.6 | 11.3 | 2.7 | 6.7 |
| Asthma/Urticaria | 23 | 34.9 | 29.6 | 1.5 | 3.8 |
| Depression screening | 6 | 37.2 | 35.8 | 0.4 | 1.0 |
| Prophylaxis for HIV | 54 | 42.6 | 30.0 | 3.6 | 9.0 |
| SSBs | 243 | 215.1 | 158.5 | 16.2 | 40.5 |

HIV: Human Immunodeficiency Viruses; SSB: sugar sweetened beverages

† Estimated rate of 4 minutes/article (15 articles/hour)

‡ Estimated rate of 5 minutes/article (12 article/hour). This does not factor in any time to resolve any conflicts.
